# The effects of indoor environmental exposures on pediatric asthma: a discrete event simulation model

**DOI:** 10.1186/1476-069X-11-66

**Published:** 2012-09-18

**Authors:** M Patricia Fabian, Natasha K Stout, Gary Adamkiewicz, Amelia Geggel, Cizao Ren, Megan Sandel, Jonathan I Levy

**Affiliations:** 1 Department of Environmental Health, Boston University School of Public Health, Boston, MA, USA; 2 Department of Environmental Health, Harvard School of Public Health, Boston, MA, USA; 3 Department of Population Medicine, Harvard Medical School and Harvard Pilgrim Health Care Institute, Boston, MA, USA; 4 Department of General Pediatrics, Boston Medical University School of Medicine, Boston, MA, USA

**Keywords:** Asthma, Simulation, Indoor, Housing, Air pollution, Lung function, Allergen, Green building

## Abstract

**Background:**

In the United States, asthma is the most common chronic disease of childhood across all socioeconomic classes and is the most frequent cause of hospitalization among children. Asthma exacerbations have been associated with exposure to residential indoor environmental stressors such as allergens and air pollutants as well as numerous additional factors. Simulation modeling is a valuable tool that can be used to evaluate interventions for complex multifactorial diseases such as asthma but in spite of its flexibility and applicability, modeling applications in either environmental exposures or asthma have been limited to date.

**Methods:**

We designed a discrete event simulation model to study the effect of environmental factors on asthma exacerbations in school-age children living in low-income multi-family housing. Model outcomes include asthma symptoms, medication use, hospitalizations, and emergency room visits. Environmental factors were linked to percent predicted forced expiratory volume in 1 second (FEV1%), which in turn was linked to risk equations for each outcome. Exposures affecting FEV1% included indoor and outdoor sources of NO_2_ and PM_2.5_, cockroach allergen, and dampness as a proxy for mold.

**Results:**

Model design parameters and equations are described in detail. We evaluated the model by simulating 50,000 children over 10 years and showed that pollutant concentrations and health outcome rates are comparable to values reported in the literature. In an application example, we simulated what would happen if the kitchen and bathroom exhaust fans were improved for the entire cohort, and showed reductions in pollutant concentrations and healthcare utilization rates.

**Conclusions:**

We describe the design and evaluation of a discrete event simulation model of pediatric asthma for children living in low-income multi-family housing. Our model simulates the effect of environmental factors (combustion pollutants and allergens), medication compliance, seasonality, and medical history on asthma outcomes (symptom-days, medication use, hospitalizations, and emergency room visits). The model can be used to evaluate building interventions and green building construction practices on pollutant concentrations, energy savings, and asthma healthcare utilization costs, and demonstrates the value of a simulation approach for studying complex diseases such as asthma.

## Background

In the US, asthma is among the most common chronic diseases of childhood across all socioeconomic classes and is the most frequent cause of hospitalization among children after birth [1]. Higher asthma prevalence has been documented in low-income inner-city children in many cities [2-5]. A number of studies have documented relationships between asthma exacerbation and exposure to indoor environmental stressors found in residential settings, such as allergens (e.g., dust mites, cockroach allergens), air pollutants (e.g., ozone (O_3_), nitrogen dioxide (NO_2_), fine particulate matter (PM_2.5_)), and environmental tobacco smoke (ETS) [6,7]. Asthma exacerbations and related events are clearly influenced by numerous additional factors, including but not limited to access to health care, medication compliance, and rhinovirus and other infectious agents [8-10].

Because of the multi-factorial nature of asthma exacerbations, it can be challenging to design optimal intervention strategies. Studies have demonstrated public health benefits of residential interventions such as using community health workers to provide asthma education [11,12], conducting integrated pest management [13,14], or multi-factorial indoor interventions [15]. It is difficult to make generalized conclusions about intervention efficacy, because of significant differences in context and risk factors for various populations, as well as the possibility that intensive interventions using community health workers have significant social support components that provide benefits beyond improvements in the physical environment [16]. A recent systematic review by the Centers for Disease Control and Prevention (CDC) found that multi-trigger, multi-component, home based environmental interventions for children were effective at improving asthma quality of life and productivity, but did not specify which components were essential elements [17].

Indoor environmental interventions are also prone to complex tradeoffs among pollutants, as interventions that influence ventilation can have opposing effects on indoor and outdoor sources, and interventions that address pests can lead to increased pesticide exposures if not designed and implemented appropriately [18]. For example, higher NO_2_ concentrations tend to be measured indoors in homes with gas stoves [19,20], indicating that improved venting of gas stoves or increased ventilation in general will reduce NO_2_ concentrations. However, increasing general ventilation increases indoor NO_2_ concentrations from outdoor sources, particularly in urban settings with high traffic.

Simulation modeling can be a valuable tool for evaluating intervention strategies across a range of outcomes, especially in the presence of significant tradeoffs. In this context, simulation modeling refers to a systems science approach involving modeling of a complex system that evolves over time given changes in state variables that occur at defined points in time [21]. Such models have been used for a number of health policy analyses such as evaluating alternative interventions for schizophrenia [22], malaria [23] diabetes [24], and breast cancer [25,26]. In spite of the flexibility of a simulation modeling approach and its applicability to a multi-exposure setting for a complex multi-factorial disease such as asthma, modeling applications in either environmental exposures or asthma have been limited to date.

Prior modeling work in asthma has been limited to Markov state-transition models. These models have focused on either the adult population or interventions involving medication adherence alone [27-29]. While these studies provided valuable insight about medication cost-effectiveness, Markov state-transition models are limited given their inability to track individuals, take account of multiple individual attributes, and capture interactions and non-linear effects. By definition, Markov models are memory-less, and the probability of transitioning between states does not depend on prior history within the simulation [30]. In contrast, asthma has been characterized as a classic example of a dynamic and non-linear disease with numerous influential factors [31], for which alternative modeling approaches may be more informative.

In this article, we provide the conceptual design and parameterization for a discrete event simulation model of pediatric asthma, focusing on the efficacy of indoor environmental interventions in low-income multi-family dwellings. These residences are characterized by having smaller living spaces and high occupant density, as well as an increased prevalence of peeling paint, indoor water leaks, and structural deficiencies that provide points of entry for cockroaches and other pests [18]. The model begins with a baseline population of high-risk children – characterized by demographic, residential, and behavioral factors. For each household, we modeled indoor environmental concentrations of multiple contaminants for fifty thousand simulated households across a variety of conditions. Based on literature syntheses, these concentrations are used to predict the percent predicted forced expiratory volume in one second (FEV1%), which in turn determines the probability of asthma exacerbations and health care utilization. To establish model credibility, we compared outputs with the published literature. As model evaluation is an iterative process, we will continue this as new data come to light. Finally, we conduct a simulation experiment with a simple hypothetical intervention to illustrate the insights available through this model approach, and discuss the strengths and weaknesses of our model structure.

## Methods

### Overview

**Figure 1:**
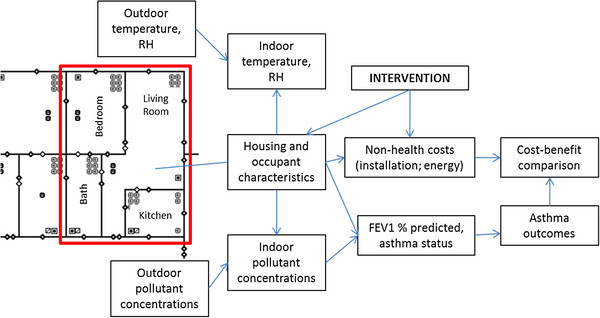
**Schematic of discrete event simulation model showing an apartment within a multi-family building from CONTAM software [**[76]**], and relationships between model inputs.**

We designed the discrete event simulation to model children’s exposure over time to environmental factors and their risk of having an asthma event on a daily basis. We modeled health outcomes by first estimating children’s exposure to indoor combustion pollutants, allergens, and other risk factors and using these factors to predict daily changes in lung function (FEV1%), as done elsewhere in the context of policy models for asthma medication [27-29]. Children were assigned a baseline FEV1% which was affected by daily random variation as well as external conditions including pollutant exposures. The daily risk of asthma outcomes (symptoms, oral steroid use, emergency room (ER) visits, hospitalizations) was calculated based on changes in FEV1% (Figure 1). The model parameters, described and justified in detail below, are summarized in Additional file 1.

The model, built in R (R 2.12.1, The R foundation of Statistical Computing), generates an ensemble or cohort of children and their associated households that have characteristics typical of Boston public housing. We used the stream package for random number generation. We used the Scientific Computer Facilities at Boston University to simulate fifty thousand children individually over 10 years, or until they turned 18 years of age. Individual results were aggregated to obtain results for the cohort.

### Study population and housing characteristics

The target simulated population was children living in low-income multi-family housing consistent with public housing residents, a population with high asthma prevalence and severity [32-34] – a community survey in two Boston Housing Authority developments found pediatric asthma prevalence of 22% [35], compared to 8.5% in the general population [36]. Inputs describing demographic and housing characteristics were drawn from studies in Boston public housing and similar settings and are presented in Table 1. In the case of age, gender, apartment level, and type of smoker (light versus heavy), values were uniformly distributed across the simulated population. The remaining characteristics obtained from studies in Boston public housing were either derived from a community-based survey administered to 53 households in one development [32], or from an asthma intervention study enrolling 78 children from 61 households in 3 developments [34]. We simulated fifty thousand children to ensure an ability to detect changes in relatively infrequent asthma events (like hospitalizations) associated with changes in a single environmental risk factor.

**Table 1:** Baseline occupant and household characteristics of a simulated population of low-income asthmatic children

**Occupant characteristics**	** Simulated population values**
Gender	50% male
Age	6-17, uniformly distributed
Race [34]	49% White
	25% African American
	15% Latino
	11% Asian
Own a gas stove[102]	89%
Use the stove for supplemental heating in winter [32]	38%, assuming that supplemental heat was turned on only on days when the 24-hour average outdoor temperature was below 32°F
Below average housekeeping (vs. average or above average housekeeping) ^a^[39]	25%
Current smoker in the house[33]	34%
Among smokers, % heavy vs. light smoker ^b^	50%
**Housing characteristics**	
Apartment level (upper 4^th^ floor/lower 1^st^ floor)	50%
Leakiness category ^c^ I	20%
[19] II	50%
III	30%
Functioning kitchen and bathroom fan [102]	13%
Houses with holes in walls/ceiling [39]	73%

^a^ Housekeeping = degree of cleanliness and clutter in the apartment, based on visual inspection.

^b^ Heavy smoker smoked one pack per day, light smoker smoked a ½ pack per day.

^c^ Leaky categories based on wall infiltration rate, where category I, II, and III had infiltration values of 0.0177, 0.053, and 0.0722 in^2^/ft^2^ respectively.

While we lacked sufficient symptom and severity data to apply the National Heart, Blood and Lung Institute (NHLBI) classification guidelines for managing asthma [8], we wanted to characterize asthma severity to determine the medications that each child would typically be prescribed. We therefore used the FEV1% cutoffs that correspond with severity classification for persistent asthmatics (> 80% for mild, 60-80% for moderate, and < 60% for severe), and used these to determine the prescribed medications. Intermittent asthmatics were excluded from the simulation because of the limited environmental literature on intermittent asthmatics – inclusion criteria for most epidemiology and environmental studies require having persistent asthma. Because of our focus on persistent asthmatics above the age of 5, many of whom are likely sensitized to one or more allergens, we assumed that asthma remained throughout childhood (i.e., no remission). Time-varying characteristics included age, indoor and outdoor NO_2_ and PM_2.5_, outdoor temperature, indoor and outdoor relative humidity (RH), cockroach allergen, mold exposure (defined by “damp” housing), daily random variation in baseline FEV1%, and changes in FEV1% due to all risk factors.

### Lung function (FEV1% predicted)

Lung function was modeled using forced expiratory volume at 1 second expiration (FEV1), a measure that is obtained by spirometry. FEV1 is generally thought to reflect airway obstruction, as observed in asthma and chronic obstructive pulmonary disease (COPD). The values of FEV1 are generally translated into a percentage of predicted FEV1 based on age, race, and gender (FEV1%), and are thought to be normal if ranging from 80 to 120% of predicted, given variations in effort by the patient or administration of the test itself. There is sufficient evidence of the relationship between FEV1% and both environmental exposures and asthma outcomes, and FEV1% is a key predictor of asthma morbidity within evidence-based national guidelines for asthma care [8]. Further, this metric has previously been used in simulation modeling analyses of asthma interventions [27-29]. Other measures of lung function, including the ratio of FEV1 to forced vital capacity (FVC), peak expiratory flow (PEF), and forced expiratory flow from the 25^th^ percentile to the 75^th^ percentile of FVC (FEF25-75%), either had an insufficient literature examining the relationship with key environmental exposures and/or were not robust measures of asthma severity. Although some investigators have shown weak correlations between FEV1% and asthma events [37,38], we considered it to be the most robust and interpretable predictor available.

The distribution of baseline FEV1% was derived using spirometry data for children with persistent asthma who participated in the Healthy Public Housing Initiative (HPHI), an intervention study focused on pediatric asthmatics living in multi-family public housing developments in Boston [13,14,19,39]. For FEV1% measurements taken prior to HPHI environmental interventions, mean baseline FEV1% was 88.4% (SD = 11.6%). However, this distribution reflects the likely cumulative effect of several environmental and socioeconomic factors present within the HPHI cohort, including indoor environmental influences. Thus, if we assumed that this distribution corresponded with a ‘no-exposure’ scenario and subsequently observed further reductions in FEV1% given the simulated exposures in our study, we would be systematically biased in our characterization of asthma severity. To address this issue, we reset the mean baseline value but kept the relative FEV1% distribution. We first ran our simulation model with the HPHI FEV1% distribution and determined the mean decrease in FEV1% attributable to all environmental exposures in comparison with a no-exposure scenario (decrease = 19.8%). We then approximated the no-exposure scenario by increasing all baseline FEV1% values by 19.8%, with a maximum allowed FEV1% of 120% to avoid findings which might be inconsistent with normal pulmonary function test results. We subsequently simulate the influence of multiple environmental exposures and medication use on FEV1% and evaluate the resulting distribution, as described in more detail below.

To characterize day-to-day variation in FEV1% within individuals, we constructed a distribution of daily variability using data collected from the HPHI study. We averaged the variability of individual repeated spirometry measures for 49 children pre-intervention over multiple testing sessions in the year, with each session including at least one week of twice-daily spirometry. A total of 110 testing sessions were recorded, with an average of 10 spirometry measurements included per child per testing session, and each pre-intervention spirometry session including at least one week of twice-daily spirometry. The mean variation between measurements within sessions was 10%, which includes random variation and variation due to environmental effects. Our objective is to include only random variation, and there are no formal analytical approaches available to determine how much of the 10% was random versus due to environmental effects. For the simulation, we assumed daily random variation of 5%, half of that observed in HPHI. Thus, daily FEV1% was assigned by drawing a value from a normal distribution, with a mean value of the baseline FEV1%, and a standard deviation of 5%.

Long term decline in FEV1% was determined based on coefficients presented by O’Byrne et al. (Table 2, [40]), where children began the simulation with a typical yearly decline in FEV1% (−0.8% for 5–10 year olds and 0% for 11–17 year olds). These values were relevant to both those who took corticosteroids and those who did not. In theory, all persistent asthmatics should be prescribed corticosteroids or other controller medications according to NHLBI clinical guidelines, but studies have found gaps related to health care access, compliance, miscommunication between providers and patients, misunderstanding of asthma as a chronic vs. acute disease, and other factors. For our study, we therefore use outputs from O’Byrne et al. to represent asthmatics with appropriately prescribed medications (termed “compliant”) and those without (termed “non-compliant”). Long term decline coefficients were modified by the occurrence of a severe asthma-related event (SARE), based on the definition of SARE and coefficients presented by O’Byrne et al. (Table 2, [40]). In our simulation a SARE was defined as a hospitalization or ER visit. In 5–10 year olds, when a SARE occurred, lung function decreased by −2.1%/year in non-compliant children and remained at −0.8%/year in compliant children. In 11–17 year olds, when a SARE occurred, lung function decreased by −1%/year in non-compliant children and −0.3%/year in compliant children. These yearly rates were converted to daily rates and applied to the daily FEV1% on the day after the SARE occurred. Children were allowed to return to pre-SARE FEV1% decline rates if they remained SARE-free for 3 years, the time period used in the O’Byrne study.

**Table 2:** **Asthma medication prescription and usage as a function of FEV1%, based on NHLBI guidelines given asthma severity classification**[8]

***FEV1%***	*** Prescription***	*** Usage***
>80%	Albuterol	Take on days with asthma symptoms
	One controller medicine	Daily
60-80%	Albuterol	Take on days with asthma symptoms
	Two controller medicines	Daily
<60%	Albuterol	Take on days with asthma symptoms
	Three controller medicines	Daily

Following this approach, mean FEV1% at the end of a 10-year simulation for 50,000 asthmatic children was 84.9% (SD = 11.4%, range: 51-118%), indicating that we have reasonably represented the underlying distribution of spirometry values from HPHI, with deviations related to modeled variability in exposures not explicitly considered in the original distribution.

### Asthma medication and compliance

Medication usage is both a key outcome variable influenced by the frequency of asthma events and a potential modifier of the effect of environmental exposures on health outcomes. The probability of a child having a prescription for controller medications was determined from HPHI data. A prior publication [41] gave the percentage of children in each severity category (mild, moderate, or severe persistent) who reported being prescribed at least one controller medication. However, as our model does not have sufficient information to formally classify severity, we reanalyzed raw data from HPHI to estimate the relationship between FEV1% and the probability of being prescribed a controller medication. We used SAS (Proc Logit, version 9.1, SAS Institute Inc., Cary, NC) to calculate the odds of being prescribed asthma medication, and converted the odds ratio to a probability estimate. The resulting probability equation was:

$$P_{\mathrm{med}}=\frac{\text{exp}\left(2.228-2\text{.854*FEV1\%}\right)}{1+\text{exp}\left(2.228-2\text{.854*FEV1\%}\right)}\text{,}$$

where P_*med*_ is the probability of being prescribed and reporting taking a controller medication, and FEV1% is the baseline lung function value. Of note, we are interested in both children who are not prescribed controller medications and those who are prescribed the medications but are non-adherent. Lacking data to quantify each of these components, we assume that the self-report from HPHI represents a combination of the two. Therefore, if the caregiver reported that a child was prescribed and was taking a controller medication, it was assumed the child was “compliant” with the medications and its protective effects. A child who should have, but was not prescribed a controller, was “non-compliant” according to clinical guidelines. This may have resulted in overestimating the use of controller medication, given the literature documenting failure to adhere to medication use even with a prescription [42], but reliable compliance data were not available from the HPHI study population and literature values would not be well aligned with the available data.

Children were evaluated at the end of each simulated year to determine changes in their asthma medication prescription, approximating adjustments that would happen during a yearly physical exam. At every year anniversary we compared each child’s average FEV1% during the past year to his or her FEV1% at the beginning of the year. Although severity classification and changes in medication are based on many components beyond FEV1%, we simplified this step by using the standard ranges of FEV1% associated with each severity classification [8], and changing their asthma medication prescription accordingly (Table 2).

### Linking indoor pollutant exposures and FEV1%

The model includes four contaminants that can potentially affect a child’s lung function and asthma status. We modeled two combustion pollutants – NO_2_ from gas stoves or outdoors, and PM_2.5_ from cooking, smoking, or outdoors – and two allergens (cockroach and dampness as a proxy for mold). Other common pollutants associated with asthma exacerbations such as ozone, mouse, cat, dog, and dust mite allergen were not included because we either lacked a critical mass of literature linking the exposure with FEV1%, or an ability to readily model indoor concentrations.

For each of the contaminants included in the study, we conducted a systematic literature review using PubMed. We initially utilized broad search criteria described below, and subsequently reviewed each article for its interpretability and applicability to our discrete event simulation model. For example, for NO_2_ and PM_2.5_, studies needed to utilize exposure metrics that could be constructed from our indoor air quality modeling (described below), and needed to include asthmatic children who were reasonably representative of our simulated population. For cockroach allergens, articles were included if they measured allergen levels in the household dust of study participants, given the difficulty in linking physical environmental measurements to measures such as IgE expression. For studies of mold and/or moisture, articles were included if they included exposure characterization that would be feasible within our exposure modeling, which focused on moisture/mold characterization given RH outputs.

For PM_2.5_ and NO_2_, we conducted a joint literature review given a desire to avoid double-counting of effects (i.e., with single-pollutant models not controlling for other pollutants). PubMed search terms included [“lung function” AND(PM2.5 OR particulate matter OR nitrogen dioxide OR NO2) AND(asthma OR children)], [("childhood asthma" OR “pediatric asthma”) AND("particulate matter" OR “nitrogen dioxide”)], and [asthma AND("particulate matter" or “nitrogen dioxide”) AND FEV]. These searches collectively yielded 413 publications, though with multiple duplicates.

Of these publications, 17 were sufficiently relevant to our study to merit more in-depth evaluation [43-59]. Studies were excluded for a variety of reasons, including a lack of focus on short-term changes in lung function due to short-term changes in air pollution. We eliminated 12 of these studies given issues such as the use of FEV1 rather than FEV1% as the outcome measure without sufficient data to allow for conversion [45,46,50,53-55,57-59], season-specific characterization [56], or missing quantification of values necessary for our study [47,51]. For the remaining five studies, two [43,44] were panel studies in Los Angeles, two [49,60] were studies of the same panel of children in Windsor, Ontario, and one [52] was based on children within the Inner City Asthma Study. We did not formally pool these available estimates because of the small number of applicable independent studies, as well as the significant heterogeneity in exposure metrics and statistical approaches. Instead, we chose the one study by O’Connor et al. [52] that provided multi-pollutant estimates among the full study population of children with exposure metrics that were available from our indoor air quality simulations. To ensure that this choice of study did not significantly bias our model, we conducted inverse-variance weighted pooling of the other four studies using the most comparable estimates available, and compared the results to those derived strictly from O’Connor et al. In spite of some limitations in the four studies and key differences in methods and assumptions, the pooled concentration-response unction was reasonably similar to the values from O’Connor et al. (20% higher for PM_2.5_ and 34% lower for NO_2_), providing reassurance that our choice was reasonable.

More specifically, we used coefficients reported in Table III of O’Connor et al. to estimate the effect of NO_2_ and PM_2.5_ on FEV1%. They reported a change in FEV1% at the 90^th^ percentile of NO_2_ and PM_2.5_ concentration relative to the 10^th^ percentile for exposure to a 5-day average outdoor pollutant concentration, adjusted for site, month, site-by-month interaction, temperature, intervention group and multiple pollutants (PM_2.5_, NO_2_, and O_3_). We converted the coefficients to a percent change in FEV1% per unit increase in pollutant, and divided by an infiltration factor to convert outdoor concentrations to equivalent indoor pollutant concentrations. This method was applied elsewhere to align epidemiologic effect estimates based on outdoor measurements with an indoor exposure model [61]. The infiltration factor for NO_2_ was 0.58 (average of factors reported by [20,62-64]), and 0.72 for PM_2.5_ (average of factors reported by [64-66]). Coefficients were a −0.093% (SE = 0.030) change in FEV1% per 1 ppb increase in NO_2_, and −0.077% (SE = 0.032) change in FEV1% per 1 μg/m^3^ increase in PM_2.5_.

For cockroach allergen exposure, we found 36 results using the PubMed search term [“pediatric asthma AND cockroach”] and 75 results using the search term [“childhood asthma AND cockroach”], but none of these articles met our inclusion criteria. Examination of other articles known to the authors did not provide adequate information for our application, largely due to a lack of quantitative exposure measures in residential dust, which would be necessary to model the marginal benefits of intervention strategies. One option was to use studies of IgE expression in children [67]. However, this would require us to dynamically model the association between allergen concentrations in dust and serum IgE, which would be quite complex and uncertain. The other option was to use studies that used a more relevant exposure metric but focused on adults. We opted for the latter approach, and selected an individual study with all relevant attributes but conducted in adults (asthmatics and non-asthmatics).

In this study, Weiss et al. found that log-transformed dust concentrations of Bla g 1 and Bla g 2 were both significantly associated with longitudinal FEV1 decline (ΔFEV1), with multiple linear regression coefficients of −194.14 mL/year and −94.83 mL/year respectively, after adjusting for age, initial FEV1, ever smoking, and Der p 1, Der f 1, and Fel d 1 allergen concentrations [68]. The study did not report functions for asthmatics only, so we used values for the entire population, noting that the relationship between dust concentrations and FEV1 was not appreciably different for the non-asthmatic population than the population as a whole. We converted change in FEV1 (ΔFEV1) to change in FEV1% by dividing ΔFEV1 by FEV1 predicted, where FEV1 predicted was calculated using the NHANES equation below [69], using the average age and height reported in Table 1 of the Weiss study.

$$\begin{array}{l} \text{FEV}1_{\mathrm{predicted}}=0.554-0.013*\text{Age}-0.0002*{\text{Age}}^{2}\\ \;-0.0001*{\text{Height}}^{2}\text{,} \end{array}$$

where age = 57.5 years, and height = 174.42 cm [68]. This conversion would ideally be based on individual height and age, but lacking this information, using the average values provides a reasonable approximation given the relatively narrow age and height ranges within the study (57.50 +/− 2.58 and 174.42 +/− 2.33, respectively). The resulting yearly decreases in FEV1% per unit increase in log transformed allergen concentration (log10 U/g) were −0.055% (SE = 0.013) and −0.027% (SE = 0.007) for Bla g 1 and Bla g 2, respectively.

Articles on mold and moisture were found in PubMed using the phrases [“childhood asthma AND mold”], [“childhood asthma AND moisture”], [“pediatric asthma AND mold”], and [“pediatric asthma AND moisture”], with 88, 13, 44, and 1 articles found, respectively. Five articles [70-74] matched the inclusion criteria, but only two measured our lung function of interest, FEV1, and only one paper was a study of asthmatics [73]. If a house was characterized as damp, we used the coefficient reported in this paper to reduce FEV1% by 10.6% (SE = 4.95, 95% CI: 1.0 – 20.3). The coefficient reported in this paper was adjusted for unemployment, household income (above/below £200), respondent smoker, other smoker in house, and pet ownership.

### Indoor pollutant exposure

For NO_2_ and PM_2.5_, daily 24-hour average exposures were estimated with regression models developed using the multi-zone simulation software output from CONTAM2.4c (NIST, Gaithersburg, MD, http://www.bfrl.nist.gov/IAQanalysis), an approach described in more detail elsewhere [75]. Briefly, within CONTAM, we selected the building most typical of Boston public housing and other low-income multi-family dwellings in Boston –a building 4 stories, 1940–1969 construction, and naturally ventilated [76]. A family of 2 adults and 2 children were simulated living in each 703 square foot apartment, which included a bedroom, bathroom, living room, and kitchen (Figure 1). Sources of NO_2_ included the gas stove used for cooking, the gas oven used for supplemental heat in the winter, and outdoors. Sources of PM_2.5_ included environmental tobacco smoke, cooking, and outdoors.

Because CONTAM could not be directly linked with the discrete event simulation model across all 50,000 children on an hourly basis for 10 years, we instead constructed regression models to explain variability in CONTAM outputs from a series of runs across key factors known to influence indoor concentrations. Regression predictors for pollutant/source combinations included terms consistent with a one-compartment box model for indoor concentrations, building characteristics (floor level, air exchange rate), occupant behaviors (kitchen and bathroom exhaust fan usage), and meteorological conditions (season, outdoor RH). Regression models had good predictive power (R^2^ from 0.89 to 0.98 across models) and physical interpretability, and outputs corresponded well with literature values [75]. Based on these equations, the 24-hour concentration was updated daily in the simulation model. See Additional file 1 for equations.

Regression models for RH were calculated in a similar way using CONTAM outputs, and were used to determine the likelihood of mold growth and dampness, described below. Indoor sources of RH included occupant behaviors such as breathing, showering, cooking, and dishwashing. Our models indicated a strong effect of season, air exchange rate, and outdoor RH and temperature [75]. We constructed exposure measures to be homologous with epidemiological evidence linking dampness/mold with FEV1%, described above. Mold growth was calculated using a set of differential equations developed to model mold growth on wooden material [77] which estimate a daily mold index (M) (Table 3). The index is a function of critical RH necessary for mold growth, temperature, surface quality, time, current RH, wood species, and some constant coefficients. Research suggests that models for pine sapwood most closely approximate materials with high nutrient content, as is the case with modern building materials [78], thus we applied the pine sapwood parameters to model mold growth in our buildings.

**Table 3:** **Description of mold index developed to describe mold growth in wood**[77]

**Mold index (M)**	** Mold growth description**
0	No growth
1	Some growth detected only with microscopy
2	Moderate growth detected with microscopy (coverage more than 10%)
3	Some growth detected visually
4	Visually detected coverage more than 10%
5	Visually detected coverage more than 50%
6	Visually detected coverage 100%

All homes were assigned a mold index value of 0 at the beginning of the simulation. Each day a change in mold index (dM/dt) was calculated based on the parameters described, where if a critical RH was reached, the index would increase, and if the RH was below a critical value, the index would decrease. Once level 2 was reached, a house was categorized as “damp”, and had an effect on FEV1% as described above. This effect was reversible, that is, if the index dropped below 2 on subsequent days, the “damp” classification was eliminated, as was the effect on FEV1%. If level 4 was reached, then the house was permanently classified as “damp”, that is, even if the index dropped below 2, the house retained its “damp” value and effect on FEV1%. See Additional file 1 for equations.

For cockroach allergen, simulations of daily concentrations were infeasible, as models such as CONTAM are not applicable. Instead, distributions for Bla g 1 and Bla g 2 were calculated from HPHI data, where Peters et al. measured cockroach allergen in air, dust in bedrooms and dust in kitchens [39]. As the Weiss et al. epidemiological study used to derive the concentration-response function chose the highest concentration among kitchen, living room, and bedroom as their exposure metric, we utilized the kitchen measurements from Peters et al. 2007, which were consistently highest in HPHI. Peters presented multivariate models where cockroach allergen concentrations depended on having holes in walls and ceilings, and the degree of cleanliness and clutter in the apartment, termed housekeeping practices. We derived three distributions of cockroach allergen from the HPHI data, where homes were categorized as: 1.“with holes and below average housekeeping”, 2.“with holes and average or above average housekeeping”, and 3.“without holes and average or above average housekeeping”. Holes were defined as “open cracks or holes thicker than a dime found in the inside walls or ceilings”. Bla g 1 geometric mean concentrations were 143.5 (GSD = 3.6), 42.7 (GSD = 6.2), and 8.2 (GSD = 14.6) U/g, for each category respectively, and Bla g 2 geometric mean concentrations were 691.4 (GSD = 8.6), 117.3 (GSD = 9.0), and 21.9 (GSD = 12.5) U/g, respectively. Because the concentrations were skewed, daily cockroach allergen concentrations were drawn from the log transformed data, truncated at one SD.

Daily NO_2_, PM_2.5_, and cockroach allergen were multiplied by a factor of 0.7 to account for the time children spent inside their homes. The 0.7 factor was calculated using data in Table 15–3 of the EPA Exposure Factors Handbook [79], which lists average times spent on indoor and outdoor activities for children 6 to 8 years old. Indoor activities included sleeping, personal care, household work, eating, studying, playing, TV, and reading. We calculated the average time spent indoors, weighted for weekend and weekday differences. For the remaining time, we assumed that outdoor NO_2_ and PM_2.5_ reasonably represented exposures (lacking data on other microenvironments) and that there were no cockroach allergen exposures.

### Outcomes: asthma exacerbations and health care utilization

Asthma outcomes were computed daily for each child based on changes in FEV1% associated with environmental exposures, derived from a prior model of the association between FEV1% and asthma symptoms or serious asthma events reported by Fuhlbrigge et al. [80]. The Fuhlbrigge study reported on 407 mild to moderate asthmatic children enrolled in the placebo branch of the Childhood Asthma Management Program (CAMP), aged 5–12 years, and recruited from 8 centers around the US [81]. In that asthma study, serious asthma event referred to ER visits, hospitalizations and oral steroid bursts, which were evaluated separately from asthma symptoms. We developed functions to approximate continuous associations between FEV1% and both asthma symptoms and serious asthma events, given only categorical models in the original publication, and we utilize other data streams to determine all outcomes of interest (which were not independently reported in the original publication).

First, the frequency of asthma symptoms was characterized in Fuhlbrigge et al. (listed in that article’s Figure 1), which shows the number of episode-free days per 4-month period across four categories of FEV1% (<60%, 60-79%, 80-99%, ≥ 100%). An episode-free day was defined as “a day with an asthma diary asthma score of 0, and no report of night awakening, morning and evening peak flow >80% personal best, no albuterol use for symptoms or prednisone use, absence from school as a result of asthma, or physician contact as a result of asthma”. We focused on the number of days with symptoms to be better aligned with our model structure. To convert this into a continuous function of FEV1%, we used the estimated midpoint of each FEV1% (50%, 70%, 90%, and 110%) category and fit the following polynomial expression:

$$\begin{array}{l} {\text{P}}_{\text{symptom\_‐day}}=2.95\;\text{FEV}1{\%}^{3}-6.93\text{FEV}1{\%}^{2}\\ \;+4.68\;\text{FEV}1\%-0.27 \end{array}$$

where P_symptom_day_ is the daily probability of having a day with asthma symptoms as defined above. While the polynomial expression is clearly over determined given four categories of FEV1%, the intent was not to build a predictive model or a model for out-of-sample characterization, but simply to capture previously observed trends. The equation is valid for values of FEV1% between 0.5 and 1.2, a range of values consistent with our simulated study population.

A similar process was used to fit an equation predicting “serious asthma events”, defined in Fuhlbrigge et al. as oral steroid use, hospitalization, or emergency room visit. Table 3 of Fuhlbrigge et al. provides a multivariate regression model including the influence of FEV1% (again in four categories) as well as night awakenings and previous hospitalizations. To convert the reported odds ratios into a probability of a serious asthma event based on a continuous FEV1% scale, we first determined the baseline rate of serious asthma events and converted it to a probability of a serious asthma event. Fuhlbrigge et al. reported that their study population had a baseline rate of 0.26 serious asthma events per 4 month period, or approximately 0.0022 events per day (probability of 0.0022). Distributing this rate on a population-weighted basis following odds ratios and population numbers in Table 1 of Fuhlbrigge et al. yields daily event probabilities of 0.0068, 0.0032, 0.0022, and 0.0017 in the four FEV1% categories of decreasing severity. Fitting a polynomial expression to these values leads to a resulting equation of:

$$\begin{array}{l} {\text{P}}_{\text{serious event}}=-0.045\text{FEV}1{\%}^{3}+0.1277\text{FEV}1{\%}^{2}\\ \;-0.1224\text{FEV}1\%+0.0417 \end{array}$$

Where P_serious event_ is the daily probability of having a serious asthma event. As previously, the equation is valid for values of FEV1% between 0.5 and 1.2. P_serious event_ was multiplied by a season factor which accounted for residual variation due to seasonally dependent variables such as respiratory virus infections and outdoor allergen concentrations [9,82-85]. The season factors were 1.11, 0.60, 1.23, and 1.05 for spring, summer, fall, and winter respectively, and were estimated from monthly ER utilization raw data collected in the Quality Improvement study between 2006 and 2008, using Boston Medical Center Health Net Plan (BMCHP) data [86]. Similar trends were observed in a 23-year survey of asthma hospitalizations in Canada (Figure 1A,[87]).

Based on data published in the Fuhlbrigge study, if a child had a hospitalization due to asthma in the previous 12 months , their probability of having a serious asthma event increased (Table 3, [80]). We calculated this multiplicative factor following the same process described above, with the resulting polynomial equation:

$$\begin{array}{l} {\text{O}}_{\text{hospit}}=-45.7\text{FEV}1{\%}^{3}+129.7\text{FEV}1{\%}^{2}\\ \;-124.4\text{FEV}1\%+42.4 \end{array}$$

where O_hospit_ is the increased odds of having a serious asthma event given an asthma hospitalization in the last 12 months, and was equal to 1 if no hospitalization had occurred.

While the P_serious event_ equation provides a robust expression for serious asthma events, data are not provided in Fuhlbrigge et al. to determine the relative distribution among these events, a critical component for policy analysis. To approximate this distribution, we used asthma statistics reported by the Centers for Disease Control and Prevention. We assumed that these average hospitalization and ER visit rates apply to the population from the Fuhlbrigge study, and that the odds ratios from Table 3 apply to each individual serious asthma related event, allowing us to create distributions of hospitalization rates and emergency room visits proportional to those calculated above for asthma events. The remaining events are presumed to be oral steroid bursts.

For hospitalization, we estimated a baseline hospitalization rate due to asthma of 0.023 hospitalizations per year per asthmatic child, combining the National Hospital Discharge Survey [88] which reports an asthma pediatric hospitalization discharge rate of 24.8 per year per 10,000 children under 15 years of age and the National Health Interview Survey [89] which reports that 10.7% of children between 5 and 11 years have asthma. This was similar to the approach used by the US Environmental Protection Agency to characterize baseline asthma hospitalization rates for regulatory analyses [90]. For this model we used more recent data.

We constructed a polynomial equation to predict the daily probability of hospitalization based on FEV1% using the approach described above, with the resulting equation:

$$\begin{array}{l} P_{\text{hosp}}=-0.0013\;\text{FEV}1{\%}^{3}+0.0037\;\text{FEV}1{\%}^{2}\\ \;-0.0036\;\text{FEV}1\%+0.0012 \end{array}$$

where P_hosp_ includes direct hospitalizations and transfers from the ER to the hospital.

For ER visits, we used an estimated emergency department visit rate of 0.1 per year per asthmatic [91] to build a similar equation, where the daily probability of going to the ER is:

$$\begin{array}{l} P_{\text{ER}}=-0.0057\;\text{FEV}1{\%}^{3}+0.0162\;\text{FEV}1{\%}^{2}\\ \;-0.0155\;\text{FEV}1\%+0.0053 \end{array}$$

Because 8% of ER visits result in hospitalization and are already accounted for in P_hosp_, we multiplied P_ER_ by 0.92 so as not to overestimate ER visits [92].

Oral steroid bursts were estimated by subtracting P_hosp_ and P_ER_ from P_serious event_.

## Results

### Model outcomes and evaluation

Using the population baseline characteristics we simulated 50,000 children over 10 years. Average yearly health outcomes stratified by FEV1% are presented in Table 4. As expected, children with lower lung function had a higher incidence of days with asthma episodes and severe asthma events. Average health outcome rates across the total simulated population align closely with our baseline model inputs drawn from the literature: 0.023 hospitalizations/year [88,89], 0.1 ER visits/year [91], 0.78 serious events/year [80]. The percentage of days with asthma symptoms per year was 41%, averaged across all simulated children. At the end of the simulation, 67% of the children had FEV1% > 80%, 32% had FEV1% between 60% and 80%, and 1% had FEV1% < 60%. This distribution is consistent with what has been observed in the field [93].

**Table 4:** Healthcare outcomes from a baseline simulation of 50,000 asthmatic children over 10 years

	***Asthma events per child per year***					
***FEV1% category***	***Days with asthma symptoms (SD***^***a***^***)***	***Serious asthma events (SD)***	***ER visits (SD)***	***Hospitalizations (SD)***	***Oral steroid bursts (SD)***	***Number of children***
> 80%	141 (11.1)	0.79 (0.80)	0.09 (0.24)	0.02 (0.11)	0.68 (0.76)	33,363
60%-80%	165 (10.4)	1.2 (0.8)	0.13 (0.19)	0.03 (0.09)	1.0 (0.7)	15,973
< 60%	183 (4.4)	2.3 (1.2)	0.22 (0.21)	0.05 (0.1)	2.0 (1.1)	664
Across all categories	149 (165)	0.94 (0.85)	0.11 (0.23)	0.026 (0.10)	0.81 (0.79)	50,000

^a^*SD* = standard deviation.

As a measure of disease progression, we found that 13.2% of children dropped to a more severe asthma classification over the 10 year simulation, solely based on a comparison of average FEV1% over the previous year to FEV1% asthma severity classification guidelines. This change in severity classification would then simulate the changes in asthma medication prescription that might occur at a yearly physical checkup. Because available evidence indicates a declining or unchanging lung function for asthmatic children over time, all else being equal, children would not move to a less severe classification within our simulation. Average FEV1% decline for compliant (assigned to get a controller medication) versus non-compliant (not assigned to get a controller medication) children was 0.18% and 0.37% respectively. Of the children who were non-compliant, 15% dropped to a more severe asthma classification over the 10 year simulation, compared to 12.5% of the compliant children.

For indoor pollutants, 24 hour average indoor NO_2_ was normally distributed with a mean concentration of 54 ppb (SD = 23 ppb, range 3–140 ppb). In the literature, reported values from field studies in Boston public housing include 43 ppb (SD = 20 ppb) measured in the kitchen, and 36 ppb (SD = 17 ppb) measured in the living room [19]. Another study measured an average of 19.6 ppb (SD = 11.0 ppb, range 5.7-61 ppb) [64]. The concentrations we simulated were above those reported in field studies, likely because of the high percentage of simulated homes with gas stoves and non-operational kitchen exhaust fans. All homes in the highest NO_2_ quartile (mean = 80 ppb) had gas stoves, 99.95% had non-operating kitchen exhaust fans, and 42% had low air exchange rates (i.e. leaky category 1, as defined in Table 1). In contrast, in the lowest NO_2_ quartile (mean = 22 ppb), only 56% of homes owned a gas stove, 51% had a non-operating fan and 16% had low air exchange rates.

Indoor PM_2.5_ was lognormally distributed, with a mean of 55 μg/m^3^ (SD = 34 μg/m^3^, range 14–394 μg/m^3^). In the literature, the mean PM_2.5_ concentrations in homes where smoking was rarely reported was 20.3 μg/m^3^ (SD = 12.5, range 6.77–74.9 μg/m^3^)[64]. This did not include ETS, estimated to increase indoor PM_2.5_ concentrations between 7 and 49 μg/m^3^[65,94-97]. The PTEAM study reported indoor PM_2.5_ concentrations of 48 μg/m^3^ averaged over 178 smoker and non-smoker homes [65]. In the simulation, in the highest PM_2.5_ quartile (mean = 101 μg/m^3^), 82% of homes had light or heavy smokers, 94% had non-operating exhaust fans, and 44% had low air exchange rates. In contrast, in the lowest PM_2.5_ quartile (mean = 25 μg/m^3^), only 1% of homes had smokers, 66% had non-operating exhaust fans, and 5% had low air exchange rates.

Cockroach allergen was distributed according to housekeeping practices and the presence of holes in walls and ceilings of the home. Bla g 1 and Bla g 2 median concentrations were 180 U/g (range 90–402 U/g) and 1270 U/g (range 245–2870 U/g) for below average housekeeping and presence of holes, 66.7 U/g (range 12.9-166 U/g) and 220 U/g (range 18–476 U/g) for above average housekeeping and presence of holes, and 20.3 U/g (range 0.8-51.8 U/g) and 49.1 U/g (range 5.9-92.7 U/g) for above average housekeeping and no holes. Peters et al. reported median kitchen floor Bla g 1 concentrations of approximately 48 U/g (Figure 1), [98]) measured in a longitudinal component of HPHI, and median kitchen floor concentrations of 61.8 U/g and 198 U/g for Bla g 1 and Bla g 2 in a cross-sectional component of the same study [39]. Gergen et al. reported geometric mean Bla g 1 values of 68.7 U/g in kitchens of inner-city homes [99], and Arbes et al. [100] found a geometric mean concentration of Bla g 1 in kitchen floors to be 287 U/g.

Approximately 19% of homes were classified as “damp” at the end of a 10 year simulation, comparable to prevalence values observed in studies of Boston public housing, where Hynes et al. reported 20% of homes had observed mold growth [34], and Brugge et al. reported that 43% of residents had smelled or seen mold in their homes [32].

**Figure 2:**
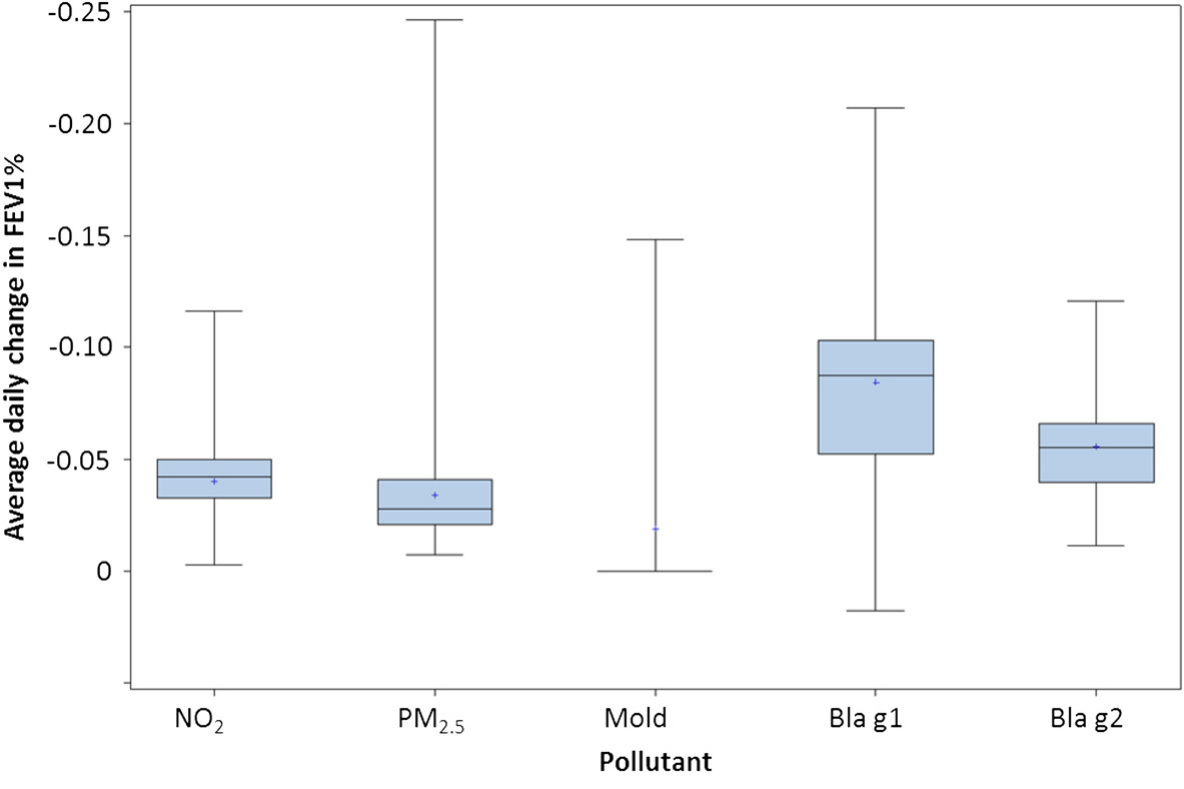
Average daily decrease in FEV1% for 50,000 simulated children over 10 years, relative to a no-exposure scenario.

As shown in Figure 2, average daily pollutant exposure decreased FEV1% by 3.4%, 4.0%, 1.9%, 8.4%, and 5.5% for PM_2.5_, NO_2_, mold, Bla g 1 and Bla g 2 respectively, relative to a no-exposure scenario, and are similar to the literature values we used as inputs. Cockroach allergen had the highest impact on FEV1%, followed by NO_2_ and PM_2.5_. In the 19% of children living in a damp (i.e. moldy) home, FEV1% decreased by 9.8%, higher than any other pollutant.

### Application example, fixing exhaust fans

We conducted a simulation experiment with a simple hypothetical intervention by simulating what would happen if the kitchen and bathroom exhaust fans were improved for the entire cohort. To evaluate this we set the exhaust fans in the kitchen and bathroom to be operational for 100% of the children, versus 13% in the baseline scenario, ran the simulation for 10 years, and compared results to the baseline 10 year simulation. In this scenario, average daily NO_2_ concentrations were reduced to 28 ppb (−48%), and PM_2.5_ concentrations were reduced to 35 μg/m^3^ (−36%). Cockroach allergen concentrations remained the same, and the percentage of homes classified as damp at the end of the simulation was 18.7%, compared to 19.0% in the baseline simulation.

In terms of health outcomes, mean daily FEV1% was 87.8% (range: 52-119%), 3% higher than the baseline scenario. Only 10.8% of children dropped to a more severe asthma category compared to 13.2% at baseline. Days with asthma symptoms, days with serious asthma events, ER visits, hospitalizations, and oral steroid use dropped 2%, 7.5%, 9.1%, 5.5%, and 8.0% respectively across all asthmatics.

For validation and evaluation purposes, we compared the magnitude of our results to relevant intervention studies as a plausibility check for our model. Unfortunately very few field studies have measured the effect of building interventions on asthma outcomes and none are directly comparable. Morgan et al. reported an 11-15% reduction in ER visits after an environmental intervention trial combining education with reducing allergen and ETS concentrations [15]. A research group in New Zealand reported a 46% drop in NO_2_ when they replaced a majority of unflued gas heaters with non-polluting heaters, with a corresponding reduction in asthma wheezing of 29% (non-significant) and a 45% reduction in sleep disturbed by wheeze (significant)[101]. Kattan et al. published on the Inner City Asthma study, showing that remediation reducing allergens to which children were specifically allergic (dust mites, passive smoking, cockroaches, pets, and rodents) decreased environmental allergens, and corresponded to a 19% decrease in unscheduled clinic visits. Since none of these interventions are directly comparable to our simulated interventions, all we can conclude is that our numbers are plausible. Further analysis comparing other interventions will be the subject of future applications of our simulation model. One approach we will take to reduce uncertainties is to focus on the marginal changes in exposures and outcomes when evaluating the effect of building interventions, rather than focusing on the absolute number of outcomes at baseline and post-intervention.

## Discussion

### Limitations

One limitation in our set of assumptions was the inability to model in more detail the cumulative effects of pollutants on FEV1%. We assumed simple additivity of effects on FEV1%, but given different mechanisms of action, the true combined effect may be different (with potential synergistic or antagonistic relationships). There is insufficient evidence in the current published literature to allow us to model these relationships. Similarly, we did not have adequate information to model the effect of non-chemical factors such as socioeconomic status, stress, or race, some of which are known to influence medication compliance or to be related to asthma outcomes. As more data become available on these relationships the model can be expanded and refined.

The equations linking FEV1% to asthma events were developed based on data from the CAMP study, which includes children with mild to moderate asthma from 8 centers across the US [81]. Thus there are some limitations in interpreting the results for the severe asthmatics as classified in our model, as well as likely differences in relationships between the CAMP children and our simulated low-income children living in multi-family housing. It is possible that using the CAMP data underestimates the severity of the response compared to lower-income populations. The same is true for our baseline estimates of asthma outcomes, which were based on national databases or the CAMP study.

The model as parameterized here is specific to multi-family housing with resident characteristics of inner-city Boston residents. Because the model relies heavily on data from the HPHI project, which involved a small number of households from a few housing developments in Boston, the generalizability of our numerical results is constrained to housing developments with similar construction (i.e. multifamily buildings) and heating systems. High rise buildings and buildings with different heating systems would require separate analysis. However, we know that this setting is relevant to many low-income urban populations where asthma prevalence is elevated and associated housing-related risk factors are also common.

The asthma medication module is an oversimplified version of what happens in the real world with respect to asthma severity classification, asthma medication prescription, and adherence. We classified asthmatics solely based on FEV1%, for the purpose of determining medications, although severity classification is far more complex [8], and medication prescription and adherence are influenced by many factors, including asthma status, access to health-care, income status, emotional status, and social experience [102]. Also, the model does not incorporate daily symptoms and level of asthma controls, and how this influences medication use, or cases where symptoms worsen over time prior to becoming a severe attack. Future work should include developing more complex models of asthma medication use which takes into account these many factors.

### Generalizability

In order to extend this framework to other settings and populations, changes in the current model can be implemented by modifying any of the parameters presented in Table 1 and elsewhere (e.g., Additional file 1), which include resident characteristics and behavior. That said, our findings are likely robust to a number of basic demographic assumptions. Even the number of residents in the unit would have a small influence on our findings given our model structure, within a reasonable range of values, so the quantitative conclusions readily extend to one-adult households.

Changes in building type or building characteristics can be implemented by selecting a different building plan from the 209 buildings available in CONTAM [76] and repeating the modeling process described, making the modeling approach generalizable.

Potential applications of this model include evaluating the effect of building construction and public housing policy changes on pollutants and asthma, evaluating green building practices on indoor air quality and health of residents in remodeled homes, and conducting cost-benefit analyses comparing energy savings to cost of healthcare utilization.

## Conclusions

We developed a discrete event simulation model of pediatric asthma that can be used to evaluate the effect of building interventions on multiple air pollutants and allergens, as well as on healthcare (asthma attacks, hospitalizations, ER visits) and asthma medicine utilization. This work presents a novel framework linking environmental exposures to FEV1% and pediatric asthma outcomes, can be expanded to any housing type, and can be refined as more data becomes available regarding the different relationships. The model can help determine health-optimal strategies as buildings are renovated or constructed, and can be used to consider a variety of environmental and non-environmental interventions targeting pediatric asthma. The outputs are directly relevant to policy discussions in low-income urban communities.

## Abbreviations

BMCHP: Boston Medical Center Health Net Plan; CAMP: Childhood Asthma Management Program; CDC: Centers for Disease Control and Prevention; COPD: chronic obstructive pulmonary disease; ER: emergency room; ETS: environmental tobacco smoke; FEF25-75%: forced expiratory flow from the 25^th^ percentile to the 75^th^ percentile of FVC; FEV1: forced expiratory volume in 1 second; FEV1%: percent predicted forced expiratory volume in 1 second; FVC: forced vital capacity; HPHI: Healthy Public Housing Initiative; NHLBI: National Heart, Lung and Blood Institute; NO_2_: Nitrogen dioxide; O_3_: Ozone; PEF: Peak expiratory flow; PM_2.5_: Particulate matter <2.5 μm; ppb: Parts per billion; RH: Relative humidity; SARE: Severe asthma-related event; SE: Standard error.

## Competing interests

The authors declare that they have no competing interests.

## Authors' contributions

NKS, MS, JIL, GA, and MPF participated in the design of the study, and defined parameters used in the model. AG researched literature and helped define parameters used in the model. CR developed the initial discrete event modeling code. MPF wrote the modeling program and drafted the manuscript. All authors read and approved the final manuscript.

## Supplementary Material

Additional file 1 Input distributions and equations for discrete event simulation model. Click here for file
