# Supplementary material for: The effects of indoor environmental exposures on pediatric asthma: a discrete event simulation model
Source: Environ Health. 2012 Sep 18;11:66. doi: 10.1186/1476-069X-11-66 (PMC3527278; doi:10.1186/1476-069X-11-66)
Supplement: Additional file 1 — Input distributions and equations for discrete event simulation model. [file 1476-069X-11-66-S1.docx]

**Supplement 1. Input distributions and equations for discrete event simulation model**

| **TABLE 1. MODEL INPUTS FOR DISCRETE EVENT SIMULATION MODEL** | | | | |
| --- | --- | --- | --- | --- |
| **FEV1% distribution & daily variation** | | | | |
|  | *Mean* | *Standard deviation* | *Distribution* | *Reference* |
| Baseline FEV1% | 88.4% | 11.6 | normal | raw data from HPHI study (n=48) |
| FEV1% daily difference | 5% | raw data from HPHI study (n = 110 individual week long spirometry sessions, with an average 10 observations/session). Total variability (random + environmental effects) was 10%, we assumed 5% was random variability | | |
| **Long term changes in FEV1%** | | | | |
| Yearly ΔFEV1% for 5-10 year old asthmatics ([O'Byrne, Pedersen et al. 2009](#_ENREF_15)) | | | | |
| Without SARE^a^ in last 3 years, compliant and non-compliant | -0.8% |  | | |
| With SARE^a^ in last 3 years, compliant | -0.8% |  |  |  |
| With SARE^a^ in last 3 years, noncompliant | -2.1% |  |  |  |
| Yearly ΔFEV1% for 11-17 year old asthmatics ([O'Byrne, Pedersen et al. 2009](#_ENREF_15)) | | | | |
| Without SARE^a^ in last 3 years, compliant and non-compliant | 0% |  | | |
| With SARE^a^ in last 3 years, compliant | -0.3% |  |  |  |
| With SARE^a^ in last 3 years, noncompliant | -1% |  |  |  |
| **Asthma medication compliance** See section E1 below | | | | |

| **Coefficients for association between FEV1% and pollutants** | | | | |
| --- | --- | --- | --- | --- |
|  | *Mean* | *Standard*  *error* | *Distribution* | *Reference* |
| ΔFEV1% per unit increase in NO_2_ (ppb) | -0.093 % | 0.030 | normal | ([O'Connor, Neas et al. 2008](#_ENREF_16)) |
| ΔFEV1% per unit increase in PM_2.5_ (ug/m^3^) | -0.077% | 0.032 | normal | ([O'Connor, Neas et al. 2008](#_ENREF_16)) |
| ΔFEV1% when house classified as “damp” | -10.6% | 4.95 | normal | ([Williamson, Martin et al. 1997](#_ENREF_22)) |
| ΔFEV1% per unit increase in log transformed Bla g 1 concentration (U/g) | -0.055% | 0.013 | normal | ([Weiss, O'Connor et al. 1998](#_ENREF_21)), see section E4 below |
| ΔFEV1% per unit increase in log transformed Bla g 2 concentration (U/g) | -0.027% | 0.007 | normal | ([Weiss, O'Connor et al. 1998](#_ENREF_21)), see section E4 below |
| **Indoor pollutant concentrations** | | | | |
| PM_2.5_ and NO_2_ | Estimated using regression models, see section E2 below | | | |
| Mold growth or dampness | Estimated using differential equations, see section E3 below | | | |
| Cockroach allergen | *Geometric mean* | *Geometric standard deviation* | *Distribution* | *Reference* |
| Bla g 1 in houses… | | | | |
| a) with holes in walls and below average housekeeping | 143.5 U/g | 3.6 | lognormal | raw data from  ([Peters, Levy et al. 2007](#_ENREF_19)) |
| b) with holes in walls and average or >average housekeeping | 42.7 U/g | 6.2 | lognormal | raw data from  ([Peters, Levy et al. 2007](#_ENREF_19)) |
| c) without holes and average or >average housekeeping | 8.2 U/g | 14.6 | lognormal | raw data from  ([Peters, Levy et al. 2007](#_ENREF_19)) |
| Bla g 2 in houses… | | | | |
| a) with holes in walls and below average housekeeping | 691.4 U/g | 8.6 | lognormal | raw data from  ([Peters, Levy et al. 2007](#_ENREF_19)) |
| b) without holes in walls and average or >average housekeeping | 117.3 U/g | 9.0 | lognormal | raw data from  ([Peters, Levy et al. 2007](#_ENREF_19)) |
| c) without holes and average or >average housekeeping | 21.9 U/g | 12.5 | lognormal | raw data from  ([Peters, Levy et al. 2007](#_ENREF_19)) |

| **Baseline rates of asthma health outcomes** | | | | |
| --- | --- | --- | --- | --- |
| Serious asthma events | 0.26 events/4 month period | | | ([Fuhlbrigge, Weiss et al. 2006](#_ENREF_8)) |
| Hospitalizations | 0.023 per year per asthmatic child | | | ([CDC 2007](#_ENREF_3); [CDC 2009](#_ENREF_4)) |
| Emergency room (ER) visits | 0.1 per year per asthmatic child | | | ([Akinbami 2006](#_ENREF_1)) |
| **Associations between FEV1% and asthma health outcomes** | | | | |
| Probability of having an asthma symptom day | | | | See section E5 below |
| Probability of having a “serious” asthma event | | | | See section E6 below |
| Probability of asthma hospitalization | | | | See section E7 below |
| Probability of ER visits | | | | See section E8 below |
| **Other factors** | | | | |
| Indoor multiplication factor (time spent indoors) | | | 0.7 | Table 15-3 of ([EPA 2009](#_ENREF_6)) |
| Seasonality factor for “serious” asthma health outcomes | | | ([Sandel 2011](#_ENREF_20)) | |
| Spring | 1.11 |  | | |
| Summer | 0.60 |  |  |  |
| Fall | 1.23 |  |  |  |
| Winter | 1.05 |  |  |  |
| NO_2_ indoor/outdoor infiltration | 0.58 | Average of infiltration rates reported by ([Monn, Fuchs et al. 1997](#_ENREF_14); [Lee, Levy et al. 1998](#_ENREF_11); [Levy, Lee et al. 1998](#_ENREF_12); [Baxter, Clougherty et al. 2007](#_ENREF_2)) | | |
| PM_2.5_ indoor/outdoor infiltration | 0.72 | Average of infiltration rates reported by ([Özkaynak, Xue et al. 1996](#_ENREF_17); [Long, Suh et al. 2001](#_ENREF_13); [Baxter, Clougherty et al. 2007](#_ENREF_2)) | | |

^a^SARE = severe asthma-related event, defined in our model as a hospitalization or ER visit

**EQUATIONS**

**E1. Probability of being prescribed and adhering to taking prescribed asthma medication (i.e. “compliant”)**

We used data from HPHI to estimate the relationship between FEV1% and the probability of being prescribed a controller medication. We used SAS (Proc Logit, version 9.1, SAS Institute Inc., Cary, NC) to calculate the odds of being prescribed asthma medication, and converted the odds ratio to a probability estimate. The resulting probability equation was:

$$P_{med}=\text{ }\frac{\text{exp(2.228-2.854*FEV1\%)}}{1+\text{exp(2.228-2.854*FEV1\%)}}$$

where: P*_med_* is the probability of reporting a controller medication

FEV1% is the baseline lung function value.

**E2. 24-hour indoor NO_2_ and PM_2.5_ concentration equations**

For NO_2_ and PM_2.5_, daily 24-hour average exposures were estimated with regression models developed using the multi-zone simulation software output from CONTAM2.4c (NIST, Gaithersburg, MD, <http://www.bfrl.nist.gov/IAQanalysis>), an approach described in more detail elsewhere ([Fabian, Adamkiewicz et al. 2011](#_ENREF_7)). Briefly, within CONTAM, we selected the building most typical of Boston public housing and other low-income multi-family dwellings in Boston –a building 4 stories, 1940-1969 construction, and naturally ventilated ([Persily, Musser et al. 2006](#_ENREF_18)). A family of 2 adults and 2 children were simulated living in each 703 square foot apartment, which included a bedroom, bathroom, living room, and kitchen. Sources of NO_2_ included the gas stove used for cooking, the gas oven used for supplemental heat in the winter, and outdoors. Sources of PM_2.5_ included environmental tobacco smoke, cooking, and outdoors. Based on the regression models developed, the 24-hour concentration of each pollutant was updated daily in the simulation model. Tables 2 and 3 show the regression equations, copied from Fabian et al.

**Table 2.** **Regression models predicting indoor NO_2_ concentrations from cooking, heating the house with the oven, and outdoors, from a database of apartments in a multi-family building simulated with CONTAM.**

| **Dependent variable: log (NO_2_ from cooking (µg/m^3^)) Model R^2^=0.89** | | | | | | |
| --- | --- | --- | --- | --- | --- | --- |
|  | Estimate (β) | Standard Error | t value | P value | Univariate R^2^ | Partial R^2^ |
| Intercept | 3.03 | 0.03 | 93.5 | <.0001 | - | - |
| Fan off | 0.98 | 0.01 | 68.8 | <.0001 | 0.60 | 0.60 |
| Box model term 1^a^ | 0.47 | 0.02 | 28.0 | <.0001 | 0.29 | 0.86 |
| AER^b^ | -0.06 | 0.01 | -9.9 | <.0001 | 0.20 | 0.87 |
| Lower level | -0.23 | 0.02 | -14.3 | <.0001 | 0.01 | 0.89 |
|  | | | | | | |
| **Dependent variable: log (NO_2_ from heating in winter(µg/m^3^)) Model R^2^= 0.98** | | | | | | |
|  | Estimate (β) | Standard Error | t value | P value | Univariate R^2^ | Partial R^2^ |
| Intercept | 3.56 | 0.03 | 106.6 | <.0001 | - | - |
| Box model term 2^c^ | 1.91 | 0.05 | 38.1 | <.0001 | 0.92 | 0.92 |
| AER^b^ | -0.11 | 0.01 | -22.2 | <.0001 | 0.82 | 0.96 |
| Lower level | -0.16 | 0.01 | -11.1 | <.0001 | 0.13 | 0.98 |
| Fan off | 0.05 | 0.01 | 4.1 | <.0001 | 0.004 | 0.98 |
|  | | | | | | |
| **Dependent variable: log (NO_2_ from outdoors(µg/m^3^)) Model R^2^=0.90** | | | | | | |
|  | Estimate (β) | Standard Error | t value | P value | Univariate R^2^ | Partial R^2^ |
| Intercept | 0.83 | 0.03 | 25.3 | <.0001 | - | - |
| Infiltration term^d^*NO_2_ out | 141.71 | 2.58 | 54.9 | <.0001 | 0.86 | 0.86 |
| AER^b^ | -0.02 | 0.01 | -3.7 | 0.0002 | 0.50 | 0.87 |
| Season: Fall | 0.16 | 0.02 | 8.7 | <.0001 | 0.13 | 0.87 |
| Season: Spring | 0.08 | 0.02 | 4.6 | <.0001 | - | - |
| Season: Summer | 0.14 | 0.02 | 7.1 | <.0001 | - | - |
| Lower level | 0.21 | 0.01 | 15.2 | <.0001 | 0.06 | 0.90 |

^a^ Box model term 1=stoveuse/(aer+kNO_2_)

^b^ AER= air exchange rate or air change rate

^c^ Box model term 2= 1/(aer+kNO_2_)

^d^ Infiltration term= p*aer/(aer+kNO_2_)

**Table 3. Regression models predicting indoor PM_2.5_ concentrations from cooking, environmental tobacco smoke (ETS), and outdoors, from a database of apartments in a multi-family building simulated with CONTAM.**

| **Dependent variable: log (PM_2.5_ from cooking (µg/m^3^)) Model R^2^=0.91** | | | | | | |
| --- | --- | --- | --- | --- | --- | --- |
|  | Estimate (β) | Standard Error | t value | P value | Univariate R^2^ | Partial R^2^ |
| Intercept | 2.95 | 0.03 | 111.3 | <.0001 | - | - |
| Fan off | 1.02 | 0.02 | 62.9 | <.0001 | 0.43 | 0.43 |
| Box model term 1^a^ | 0.24 | 0.01 | 38.5 | <.0001 | 0.43 | 0.83 |
| AER^b^ | -0.15 | 0.01 | -23.1 | <.0001 | 0.32 | 0.87 |
| Lower level | -0.38 | 0.02 | -21.0 | <.0001 | 0.01 | 0.91 |
|  | | | | | | |
| **Dependent variable: log (PM_2.5_ from ETS (µg/m^3^)) Model R^2^=0.93** | | | | | | |
|  | Estimate (β) | Standard Error | t value | P value | Univariate R^2^ | Partial R^2^ |
| Intercept | 3.64 | 0.02 | 149.0 | <.0001 | - | - |
| AER^b^ | -0.31 | 0.01 | -55.9 | <.0001 | 0.75 | 0.75 |
| Box model term 2^c^ | 0.48 | 0.01 | 44.4 | <.0001 | 0.66 | 0.86 |
| Lower level | -0.48 | 0.02 | -31.1 | <.0001 | 0.04 | 0.92 |
| Season: Fall | -0.04 | 0.02 | -2.3 | 0.0218 | 0.04 | 0.93 |
| Season: Spring | 0.04 | 0.02 | 2.0 | 0.0458 | - | - |
| Season: Summer | -0.17 | 0.02 | -8.3 | <.0001 | - | - |
|  | | | | | | |
| **Dependent variable: log (PM_2.5_ from outdoors (µg/m^3^)) Model R^2^=0.91** | | | | | | |
|  | Estimate (β) | Standard Error | t value | P value | Univariate R^2^ | Partial R^2^ |
| Intercept | 0.80 | 0.02 | 35.6 | <.0001 | - | - |
| Infiltration term^d^*PM_2.5_ out | 0.13 | 0.002 | 53.3 | <.0001 | 0.59 | 0.59 |
| Season: Fall | -0.03 | 0.01 | -2.9 | 0.0045 | 0.39 | 0.76 |
| Season: Spring | -0.21 | 0.01 | -21.3 | <.0001 | - |  |
| Season: Summer | -0.42 | 0.01 | -30.8 | <.0001 | - |  |
| AER^b^ | 0.04 | 0.003 | 14.7 | <.0001 | 0.14 | 0.76 |
| Lower level | 0.28 | 0.01 | 36.4 | <.0001 | 0.01 | 0.91 |

^a^ Box model term 1= stoveuse/(aer+kPM_2.5_) ^b^ AER= air exchange rate

^c^ Box model term 2= 1/(aer+kETS) ^d^ Infiltration term= p*aer/(aer+kPM_2.5_)

**E3. Mold growth model**

The following equations were used, and are described in detail by Hukka et al ([Hukka and Viitanen 1999](#_ENREF_10)).

where:

dM/dt = change in mold index (day^-1^)

M = mold index (unitless)

t = time (days)

tm1 = time (weeks) at which mold growth will initiate at constant RH and temp, i.e. M=1

k1,k2 = correction coefficients (unitless), where

if M<1 then

k1 = 1

k2 = 1

if M>=1 then

where

tv = time (weeks) at which there will be visible mold, ie M=3

M_max_ = largest possible value of the mold index at a given

relative humidity and temperature

**E4. Cockroach allergen equations**

We selected an individual study with all relevant attributes but conducted in adults (asthmatics and non-asthmatics). In this study, Weiss et al. found that log-transformed dust concentrations of Bla g 1 and Bla g 2 were both significantly associated with longitudinal FEV1 decline (ΔFEV1), with multiple linear regression coefficients of -194.14 mL/year and -94.83 mL/year respectively ([Weiss, O'Connor et al. 1998](#_ENREF_21)). The study did not report functions for asthmatics only, so we used values for the entire population, noting that the relationship between dust concentrations and FEV1 was not appreciably different for the non-asthmatic population than the population as a whole. We converted change in FEV1 (ΔFEV1) to change in FEV1% by dividing ΔFEV1 by FEV1 predicted, where FEV1 predicted was calculated using the NHANES equation below ([Hankinson, Odencrantz et al. 1999](#_ENREF_9)), using the average age and height reported in Table 1 of the Weiss study.

$$\text{FEV1 }_{\text{predicted}}\text{ }=\text{ 0.554-0.013 * Age-0.0002*Age}^{2}+\text{0.0001 * Height}^{2}=3.52L$$

where:

age = 57.5 years ([Weiss, O'Connor et al. 1998](#_ENREF_21))

height = 174.42 cm ([Weiss, O'Connor et al. 1998](#_ENREF_21)).

where FEV1predicted = 3.52 L (calculated with previous equation)

ΔFEV1 = -194.14 mL/year for Bla g 1, and -94.83 mL/year for Bla g 2, respectively

**E5. Probability of asthma symptom days**

The frequency of asthma symptoms was characterized in Fuhlbrigge et al ([Fuhlbrigge, Weiss et al. 2006](#_ENREF_8)) (listed in that article’s Figure 1), which shows the number of episode-free days per 4-month period across four categories of FEV1% (<60%, 60-79%, 80-99%, ≥ 100%). An episode-free day was defined as “a day with an asthma diary asthma score of 0, and no report of night awakening, morning and evening peak flow >80% personal best, no albuterol use for symptoms or prednisone use, absence from school as a result of asthma, or physician contact as a result of asthma”. We focused on the number of days with symptoms to be better aligned with our model structure. To convert this into a continuous function of FEV1%, we used the estimated midpoint of each FEV1% (50%, 70%, 90%, and 110%) category and fit the following polynomial expression:

P_symptom_day_ = 2.95 FEV1%^3^ - 6.93FEV1%^2^ + 4.68 FEV1% - 0.27

where

P_symptom_day_ = daily probability of having a day with asthma symptoms as defined above.

FEV1% = forced expiratory volume 1 percent predicted

The equation is valid for values of FEV1% between 0.5 and 1.2.

**E6. Probability of “serious asthma events”**

A similar process was used to fit an equation predicting “serious asthma events”, defined in Fuhlbrigge et al. as oral steroid use, hospitalization, or emergency room visit ([Fuhlbrigge, Weiss et al. 2006](#_ENREF_8)). Table 3 of Fuhlbrigge et al. provides a multivariate regression model including the influence of FEV1% (again in four categories) as well as night awakenings and previous hospitalizations. To convert the reported odds ratios into a probability of a serious asthma event based on a continuous FEV1% scale, we first determined the baseline rate of serious asthma events and converted it to a probability of a serious asthma event. Fuhlbrigge et al reported that their study population had a baseline rate of 0.26 serious asthma events per 4 month period, or approximately 0.0022 events per day (probability of 0.0022). Distributing this rate on a population-weighted basis following odds ratios and population numbers in Table 1 of Fuhlbrigge et al. yields daily event probabilities of 0.0068, 0.0032, 0.0022, and 0.0017 in the four FEV1% categories of decreasing severity. Fitting a polynomial expression to these values leads to a resulting equation of:

P_serious event_ = -0.045FEV1%^3^ + 0.1277FEV1%^2^ - 0.1224FEV1% + 0.0417

where:

P_serious event_ = daily probability of having a serious asthma event

FEV1% = forced expiratory volume 1 percent predicted

The equation is valid for values of FEV1% between 0.5 and 1.2. P_serious event_ was multiplied by a seasonality factor.

**E7. Probability of asthma hospitalization**

We constructed a polynomial equation to predict the daily probability of hospitalization based on FEV1% using the approach described above, with the resulting equation:

P_hosp_ = -0.0013 FEV1%^3^ + 0.0037 FEV1%^2^ - 0.0036 FEV1% + 0.0012

where P_hosp_ includes direct hospitalizations and transfers from the ER to the hospital.

Based on data published in the Fuhlbrigge study, if a child had a hospitalization due to asthma in the previous 12 months , their probability of having a serious asthma event increased (Table 3, ([Fuhlbrigge, Weiss et al. 2006](#_ENREF_8))). We calculated this multiplicative factor following the same process described above, with the resulting polynomial equation:

O_hospit_ = -45.7FEV1%^3^ + 129.7FEV1%^2^–124.4FEV1% + 42.4

where:

O_hospit_ = increased odds of having a serious asthma event given an asthma hospitalization

in the last 12 months, and was equal to 1 if no hospitalization had occurred.

**E8. Probability of ER visits and oral steroid bursts**

For ER visits, we built a similar equation, where the daily probability of going to the ER is:

P_ER_ = -0.0057 FEV1%^3^ + 0.0162 FEV1%^2^ - 0.0155 FEV1% + 0.0053

Because 8% of ER visits result in hospitalization and are already accounted for in P_hosp_, we multiplied P_ER_ by 0.92 so as not to overestimate ER visits ([DPHMA 2009](#_ENREF_5)).

Oral steroid bursts were estimated by subtracting P_hosp_ and P_ER_ from P_serious event_.

**Bibliography**

Akinbami, L. J. (2006). The State of Childhood Asthma, United States, 1980–2005. Atlanta, GA, CDC. **381**.

Baxter, L. K., J. E. Clougherty, et al. (2007). "Predictors of concentrations of nitrogen dioxide, fine particulate matter, and particle constituents inside of lower socioeconomic status urban homes." J Expo Sci Environ Epidemiol **17**(5): 433-444.

CDC (2007). National Hospital Discharge Survey. Atlanta, Centers for Disease Control.

CDC (2009). Summary Health Statistics for US Children, National Health Interview Survey. Atlanta, Centers for Disease Control.

DPHMA (2009). The burden of asthma in Massachusetts. Boston, Department of Public Health.

EPA (2009). U.S. EPA. Exposure Factors Handbook (External Review Draft) 2009 Update. Washington, DC, U.S. Environmental Protection Agency.

Fabian, P., G. Adamkiewicz, et al. (2011). "Simulating indoor concentrations of NO2 and PM2.5 in multi-family housing for use in health-based intervention modeling." Indoor Air **in press**.

Fuhlbrigge, A. L., S. T. Weiss, et al. (2006). "Forced expiratory volume in 1 second percentage improves the classification of severity among children with asthma." Pediatrics **118**(2): e347-355.

Hankinson, J. L., J. R. Odencrantz, et al. (1999). "Spirometric reference values from a sample of the general U.S. population." Am J Respir Crit Care Med **159**(1): 179-187.

Hukka, A. and H. Viitanen (1999). "A mathematical model of mold growth on wooden material." Wood Science and Technology **33**(6): 475-485.

Lee, K., J. I. Levy, et al. (1998). "The Boston residential nitrogen dioxide characterization study: classification and prediction of indoor NO2 exposure." J Air Waste Manag Assoc **48**(8): 736-742.

Levy, J. I., K. Lee, et al. (1998). "Impact of residential nitrogen dioxide exposure on personal exposure: an international study." J Air Waste Manag Assoc **48**(6): 553-560.

Long, C. M., H. H. Suh, et al. (2001). "Using time- and size-resolved particulate data to quantify indoor penetration and deposition behavior." Environ Sci Technol **35**(10): 2089-2099.

Monn, C., A. Fuchs, et al. (1997). "Particulate matter less than 10 microns (PM10) and fine particles less than 2.5 microns (PM2.5): relationships between indoor, outdoor and personal concentrations." Sci Total Environ **208**(1-2): 15-21.

O'Byrne, P. M., S. Pedersen, et al. (2009). "Severe exacerbations and decline in lung function in asthma." Am J Respir Crit Care Med **179**(1): 19-24.

O'Connor, G. T., L. Neas, et al. (2008). "Acute respiratory health effects of air pollution on children with asthma in US inner cities." J Allergy Clin Immunol **121**(5): 1133-1139 e1131.

Özkaynak, H., J. Xue, et al. (1996). "Personal exposure to airborne particles and metals: results from the Particle TEAM study in Riverside, California." J Expo Anal Environ Epidemiol **6**(1): 57-78.

Persily, A., A. Musser, et al. (2006). A collection of homes to represent the U.S. housing stock. Washington DC, National Institute of Standards and Technology. **NISTIR 7330**.

Peters, J. L., J. I. Levy, et al. (2007). "Determinants of allergen concentrations in apartments of asthmatic children living in public housing." J Urban Health **84**(2): 185-197.

Sandel, M. (2011). 2006-2008 Boston Medical Center Health Net Plan data Quality Improvement study. P. Fabian. Boston.

Weiss, S. T., G. T. O'Connor, et al. (1998). "Indoor allergens and longitudinal FEV1 decline in older adults: the Normative Aging Study." J Allergy Clin Immunol **101**(6 Pt 1): 720-725.

Williamson, I. J., C. J. Martin, et al. (1997). "Damp housing and asthma: a case-control study." Thorax **52**(3): 229-234.
